# Boswellic acids ameliorate neurodegeneration induced by AlCl_3_: the implication of Wnt/β-catenin pathway

**DOI:** 10.1007/s11356-022-20611-5

**Published:** 2022-06-06

**Authors:** Eman A. Mohamed, Hebatalla I. Ahmed, Heba S. Zaky, Amira M. Badr

**Affiliations:** 1 Department of Pharmacology and Toxicology, Faculty of Pharmacy (Girls), Al-Azhar University, Nasr City, Cairo, P.N.11754 Egypt; 2 Department of Pharmacology and Toxicology, College of Pharmacy, King Saud University, P.O. Box 22452, Riyadh, Saudi Arabia; 3 Department of Pharmacology and Toxicology, College of Pharmacy, Ain Shams University, Heliopolis, Cairo, Egypt

**Keywords:** Alzheimer’s disease, Neurodegenerative diseases, Boswellic acids, Wnt/β-catenin, Aluminum toxicity

## Abstract

Alzheimer’s disease (AD) is a neurodegenerative disease (ND) that represents the principal cause of dementia. Effective treatment is still lacking. Without prevention, Alzheimer’s disease (AD) incidence is expected to triple within 30 years. The risk increases in highly polluted areas and is positively linked to chronic aluminum (Al) exposure. Canonical Wingless-Int (Wnt)/β-catenin pathway has been found to play a considerable role in ND pathogenesis. Resins of *Boswellia serrata* (frankincense) have been used traditionally for their psychoactive activity, in addition to their memory-boosting effects. Boswellic acids (BA) are pentacyclic triterpenes. They have antioxidant, anti-inflammatory, antinociceptive, and immunomodulatory activities. This study aimed to elucidate the role of the Wnt/β-catenin pathway in BA protective activity against aluminum-induced Alzheimer’s disease. For 6 weeks, rats were treated daily with AlCl3 (100 mg/kg/i.p.) either alone or with BA (125 or 250 mg/kg PO). Results indicated that BA significantly improved learning and memory impairments induced by AlCl_3_ treatment. Moreover, BA treatment significantly decreased acetylcholinesterase levels and reduced amyloid-beta (Aβ) expression. In addition, BA ameliorated the increased expression of tumor necrosis factor-alpha (TNF-α) and interleukin-1 beta (IL-1β), inhibited lipid peroxidation, and increased total antioxidants in the brain. Indeed, BA significantly suppressed AlCl_3_-induced decrease of brain-derived neurotrophic factor, pGSK-3β (Ser 9), and β-catenin. BA (250 mg/kg) showed a significant protective effect compared to a lower dose. The results conclude that BA administration modulated the expression of Wnt/β-catenin pathway-related parameters, contributing to BA’s role against Al-induced Alzheimer’s disease.

**Graphical abstract:**

Effect of Boswellic acids on AlCl_3_-induced neurodegenerative changes. ChE cholinesterase, Ach acetylcholine, BDNF brain-derived neurotrophic factor, IL-1β interleukin-1β, TNF-α tumor necrosis factor-α

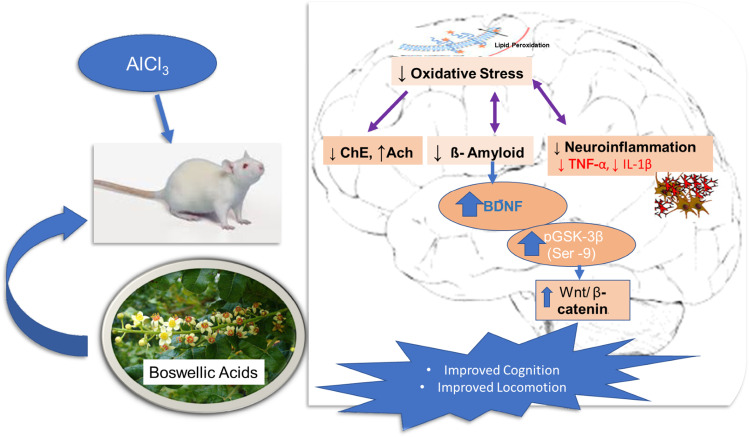

**Supplementary Information:**

The online version contains supplementary material available at 10.1007/s11356-022-20611-5.

## Introduction

The incidence of neurodegenerative diseases (NDs) is rapidly growing around the world. With the increasing age of the population, these disorders parallelly increase. Alzheimer’s disease (AD) is a ND characterized by behavioral and memory defects, accompanied by functional and cognitive impairments. AD may be considered the most offending cause of dementia in the elderly. Pathological hallmarks of AD include intracellular neurofibrillary tangles aggregated by hyperphosphorylated tau, in addition to extracellular senile plaques of beta-amyloid (Aβ) protein (Peden and Ironside 2012). Several studies have displayed different mechanisms for AD pathogenesis, including oxidative stress, amyloidogenesis theory, cholinergic dysfunction, and neuroinflammation. Although the argument, neuroinflammation and oxidative stress have been established to be the critical pathological marks of AD (Magalingam et al. 2018). AD has been linked to environmental pollution, with several pollutants implicated, such as carbon monoxide, ozone, and particulate matter (Fu and Yung 2020). Aluminum (Al) toxicity has ample evidence of being linked to AD (Colomina and Peris-Sampedro 2017).

The advancement of recognizing the molecular pathological mechanisms underlying AD is mandatory for evolving new therapeutic strategies that help prevent or halt disease progression. Increasing evidence suggests that dysregulation of the canonical Wingless-Int (Wnt)/β-catenin pathway could be embroiled in the NDs pathogenesis. Downregulation of the Wnt/β-catenin signaling cascade has been linked with AD onset and progression and synaptic stability (Jia et al. 2019b). Glycogen synthase kinase 3β (GSK-3β) is a crucial regulator of the Wnt canonical pathway. Activation of this pathway leads to the inhibition of GSK-3β activity and an increase of β-catenin activity. β-catenin is a transcriptional molecule that migrates to the nucleus and stimulates the transcription of target genes. On the contrary, if the Wnt canonical pathway is switched off, GSK-3β activity increases and stimulates β-catenin degradation. Remarkably, GSK-3β/β-catenin has been implicated in neuronal survival, neurodegeneration, and memory integration (Libro et al. 2016). Wnt/β-catenin signaling is critically associated with oxidative stress in AD (Xian et al. 2016; Libro et al. 2016; Vallée et al. 2017; Wang et al. 2019). Furthermore, GSK-3β/Wnt signaling was found to play a role in neurodegeneration in AD through the induction of inflammatory and apoptotic pathways. In addition, Wnt/β-catenin signaling regulates the expression of brain-derived neurotrophic factor (BDNF). BDNF is a vital neurotrophin that governs neuronal cells’ growth, survival, and differentiation. It also modulates cognitive functions and hinders neuroinflammation (Yang et al. 2016).

The application of traditionally used natural products to prevent and treat diseases, especially chronic diseases, has attracted much attention because of their relative safety and scientifically proven activity. Pentacyclic triterpenes are a class of compounds with multiple biological activities, among them Boswellic acids (BAs). BAs are the main constituents separated from the gum resin of *Boswellia* *serrata* (recognized as Frankincense or olibanum), such as β-boswellic acid, 3-acetyl-α-boswellic acid, 11-keto-β-boswellic acid, and acetyl-11-keto-β-boswellic acid (AKBA). The Boswellia species (frankincense) resins are well known worldwide. They have traditionally been used in folk medicine in India, China, and by Arabs; furthermore, they are used in Europe for religious rituals (Efferth and Oesch 2020). They were used in folk medicine to treat wounds and inflammatory diseases and their psychoactive effects (Byler and Setzer 2018). Boswellia resin extract suppresses the expression of proinflammatory mediators. In addition, it has antioxidant, anti-nociceptive, and immunomodulatory activities owing to its ability to target different signaling pathways, enzymes, transcription factors, as well as kinases (Al-Harrasi et al. 2019).

Boswellia resin was also utilized to boost the memory and learning activity (Hosseini et al. 2010; Jalili et al. 2014; Majdinasab et al. 2016; Ebrahimpour et al. 2017). Recently, Byler et al. concluded the potential activity of Boswellia resin in Alzheimer’s disease using a docking study, through its effect on acetylcholinesterase *(AChE)* (Byler and Setzer 2018). In addition, the anti-inflammatory and antiapoptotic properties of Boswellia resin make it a promising target against neuroinflammation, which characterizes NDs (Sayed and El Sayed 2016; Sayed et al. 2018). So, the current study hypothesized that BAs might have a neuroprotective activity against AlCl_3_-induced AD symptoms. Their effect is partially mediated via regulating the key masters of the Wnt/β-catenin pathway.

## Materials and methods

### Drugs and chemicals

AlCl_3_.6H_2_O was purchased from Sigma-Aldrich Chemical Co. (St. Louis, MO, USA). It was freshly dissolved in saline, and intraperitoneally (i.p.) injected. Capsules containing standardized *B. serrata* gum extract (65% BAs equivalent to 292.5 mg) were purchased from GNC Herbal Plus® (Pittsburgh, PA, USA) and dissolved in distilled water. All other chemicals were of the highest analytical grade.

### Animals

Forty adult male Sprague Dawley rats (180–200 g) were obtained from the Nile Co. for Pharmaceuticals and Chemical Industries, Cairo, Egypt. Animals were maintained in groups of five per cage in the animal facility of the Faculty of Pharmacy (Girls), Al-Azhar University, housed in a conditioned atmosphere at 25 ± 2 °C. They were kept on standard diet pellets (El-Nasr, Cairo, Egypt) and tap water ad libitum. The experiment was carried out by ethical procedures and policies approved by the Ethics Committee of the Faculty of Pharmacy (Girls), Al-Azhar University (approval no.137).

### Experimental design

Forty rats were arbitrarily split into four groups (ten animals per group). Group 1 (control group): rats were injected with saline (1 ml/kg, i.p.) and received distilled water orally (1 ml/kg, p.o.); group 2 (AD group): rats were daily injected with AlCl_3_.6H_2_O (100 mg/kg, i.p.) (Mohamed et al. 2021); groups 3 and 4 were treated with daily i.p. doses of AlCl3.6H2O and BAs (125 ml/kg or 250 mg/kg, orally) respectively (Ameen et al. 2017). All treatments were given daily for 6 weeks. Then, the behavioral tests were conducted after administering the last doses. Twenty-four hours after the behavioral tests, animals were euthanized, brains were insulated, and the hippocampus was separated from each brain, immediately rinsed in ice-cold normal saline (0.9% w/v), and finally homogenized in 0.1 M phosphate buffer (pH 7.4, the final concentration of 10% w/v) for further biochemical analysis.

### Methods

#### Behavioral testing

Two behavioral tests were used to assess different behavioral changes in rats. The open-field test (OFT) is a mild stressful condition helpful in detecting changes in exploratory behavior and emotionality (Cunha and Masur 1978). The Morris water maze (MWM) test represents a standard test of memory, and spatial learning in rodents, as Morris (1984) described (Morris 1984).

#### Protein estimation

According to the Bradford technique, the protein content was measured in the hippocampus homogenates (Bradford 1976) using standard bovine serum albumin.

#### Enzyme-linked immunosorbent assays

Commercially available enzyme-linked immunosorbent assays (ELISA) kits were used to define the levels of hippocampal AChE and amyloid beta-peptide 1–42 (Aβ1-42) (MyBioSource. Inc., USA, catalog no. MBS725468 and MBS726579, respectively), malondialdehyde (MDA) (lifeSpan Biosciences, Inc., USA; catalogue no. LS-F28018), superoxide dismutase (SOD), and total antioxidant capacity (TAC) (MyBioSource. Inc., USA, catalog no. MBS036924 and MBS733414_48T, respectively). ELISA kits from Cusabio Biotech Co., China, were used to measure the levels of IL-1β (catalog no. CSB-E08055r), tumor necrosis factor-alpha (TNF-α) (catalogue no. CSB-E11987r), BDNF (Boster Biological Technology Co., LTD, catalogue no. EK0308), and Beta-catenin (MyBioSource. Inc., USA, catalog no. MBS720420). In addition, pGSK-3β (Ser9) (RayBiotech, Inc., catalog no. PEL-GSK3b-S9-T) was assessed. All assays exactly followed the manufacturer’s instructions.

### Statistical analysis

All data were expressed as mean ± standard deviation (SD). Data were analyzed using a one-way analysis of variance (ANOVA). Tukey's multiple comparison test was used to assess differences between means. A significant difference was considered at the level of *P* < 0.05. Statistical analyses and plotting were performed using GraphPad Prism (ISI®, USA) software (version 5).

## Results

### Determination of behavioral changes

**Fig. 1:**
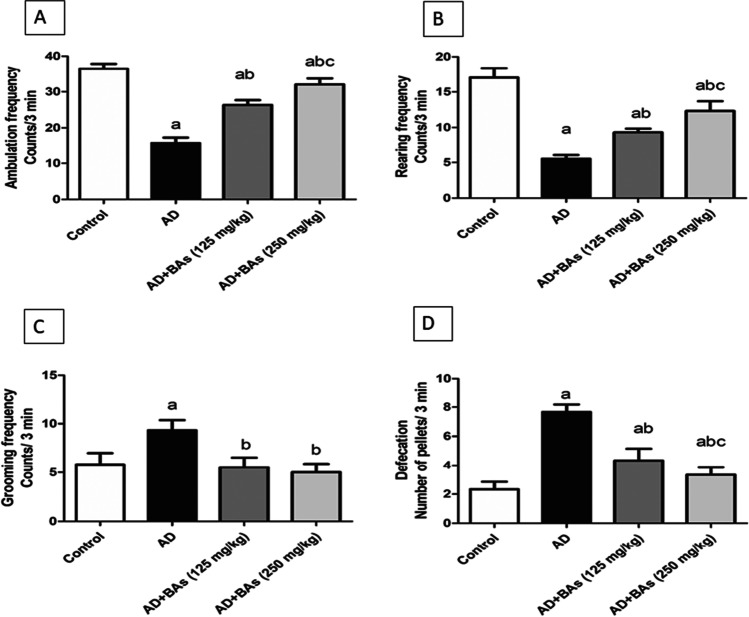
Effect of Boswellic acids on Alzheimer’s-induced behavioral alterations in the open-field test. **A** Ambulation frequency, **B** rearing frequency, **C** grooming frequency, **D** defecation. Data are mean ± SD (*n* = 8). a, b, or c: significantly different compared to the control, AD group, or BAs (125 mg/kg) group respectively, *P* < 0.05 using ANOVA followed by Tukey’s as post hoc test. AD Alzheimer’s group (AlCl_3_ (100 mg/kg)), BAs Boswellic acids

Behavioral results of OFT for different groups are shown in Fig. 1A, B, C, and D. AlCl_3_ injection significantly reduced the exploratory activity, as evidenced by the decrease in the ambulation frequency by 56.6% and the rearing frequency by 67.6% compared to the control group. Nevertheless, as a manifestation of emotionality, self-grooming and the number of pellets significantly increased after AlCl_3_ treatment to 160% and 328.6%, respectively, compared to the control rats. AlCl_3_-induced exploratory and emotional dysregulations improved considerably upon treating animals with either BAs (125 mg/kg) or BAs (250 mg/kg).

**Fig. 2:**
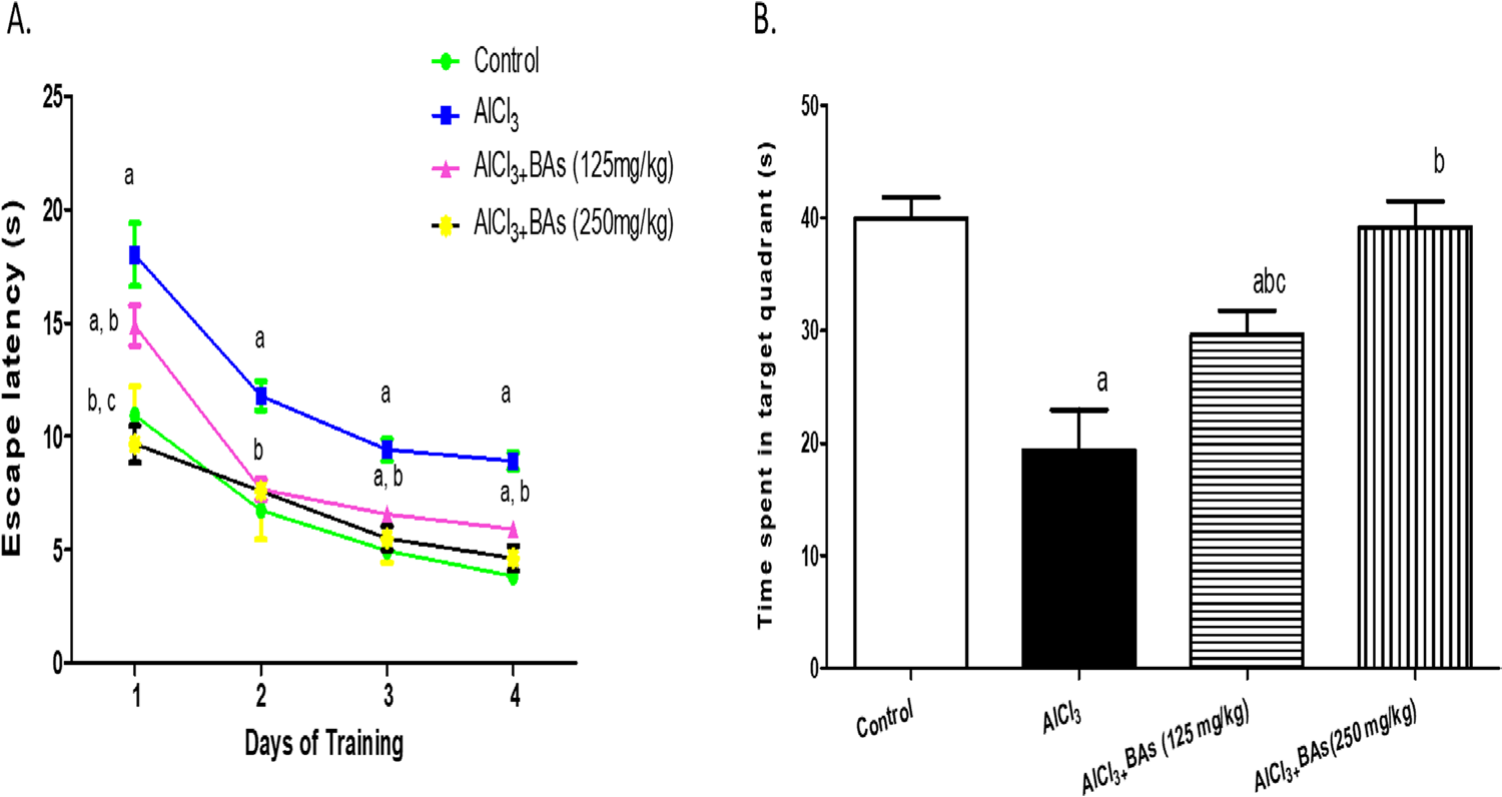
Effect of Boswellic acids on Alzheimer’s-induced behavioral alterations in the Morris Water Maze test. **A** Escape latency, **B** time spent in the target quadrant. Data are mean ± SD (*n* = 8). a, b, or c: significantly different compared to the control, AD group, or BAs (125 mg/kg) group respectively, *P* < 0.05 using ANOVA followed by Tukey’s as post hoc test. AD Alzheimer’s group (AlCl_3_ (100 mg/kg)), BAs Boswellic acids

As reported in Fig. 2A, AlCl_3_ administration caused spatial learning and memory disturbance. The efficiency of the learning ability in animals treated with AlCl_3_ was diminished, evidenced by a significant increase in escape latency from day 1 to day 4 of training by approximately 80%, 74.7%, 89.9%, and 132.6%, respectively, as compared to control values. However, concurrent treatment of animals with either AlCl_3_ + BAs (125 mg/kg) or AlCl_3_ + BAs (250 mg/kg) significantly enhanced learning ability. Rats treated with AlCl_3_ showed a significant decrease in the time spent in the target quadrant, reflecting memory deficits by approximately 51.7% compared to the control group. However, the AlCl3 + BAs (125 mg/kg) or AlCl3 + BAs (250 mg/kg) group modulate the decreased time spent where they were increased by 53.5% and 102.6%, respectively, compared to those theAlCl_3_-treated group. The higher dose of BAs produced a better enhancement in memory function (Fig. 2B).

### Assessment of AChE and Aβ1-42

**Fig. 3:**
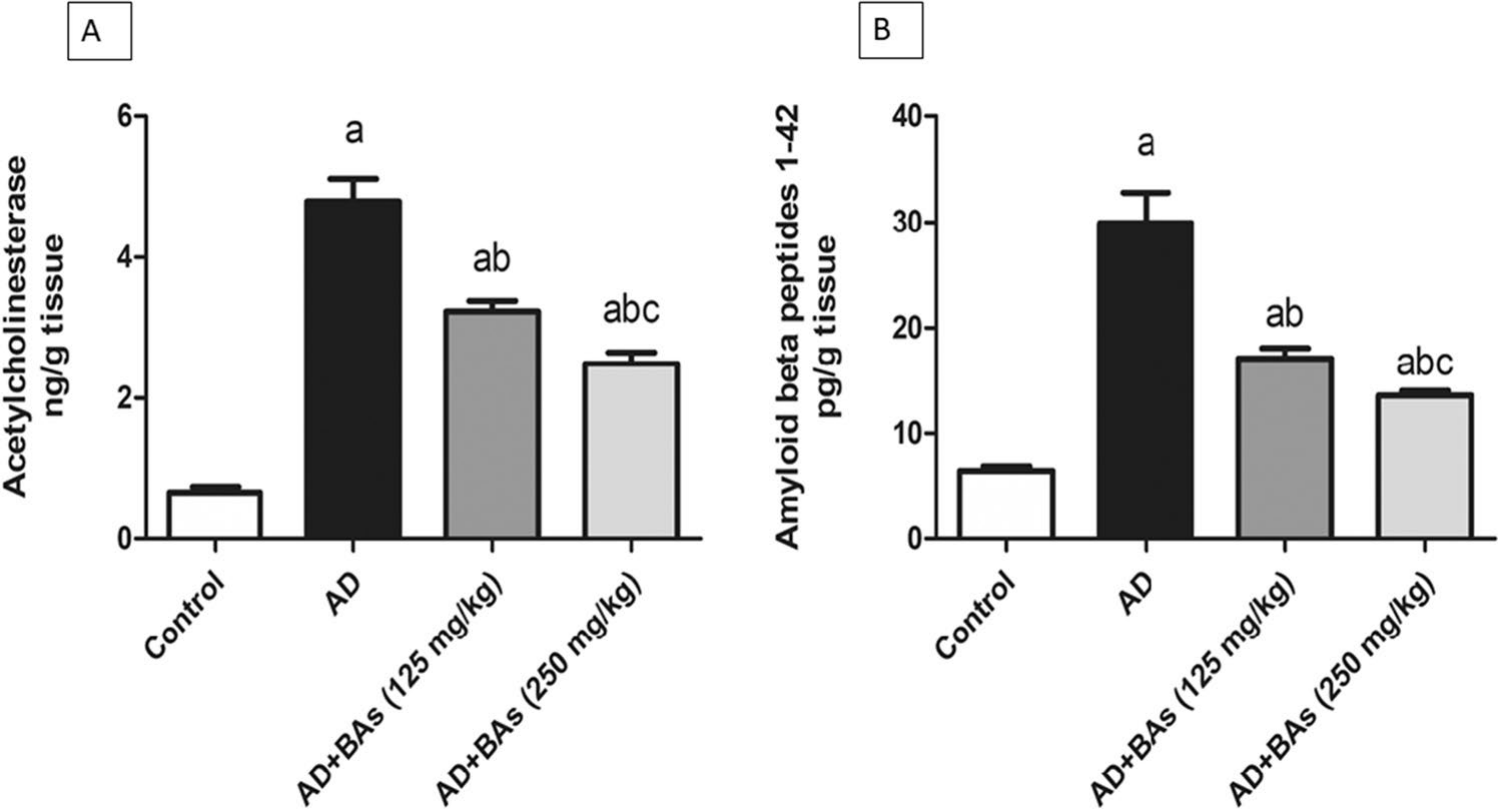
Effect of Boswellic acids on Alzheimer’s-induced alterations in the hippocampal acetylcholinesterase and amyloid-beta peptides content. **A** acetylcholinesterase, **B** amyloid-beta peptides. Data are mean ± SD (*n* = 6). a, b, or c: significantly different compared to the control, AD group, or BAs (125 mg/kg) group respectively, *P* < 0.05 using ANOVA followed by Tukey’s as post hoc test. AD: Alzheimer’s group (AlCl_3_ (100 mg/kg)); BAs: Boswellic acids

Figure 3A showed that the AChE level was markedly elevated in the AlCl_3_-treated group reaching 717.8% compared to the control group. Concurrent treatment of animals with either AlCl_3_ + BAs (125 mg/kg) or AlCl_3_ + BAs (250 mg/kg) significantly decreased AChE levels by 32.6% and 48.1% respectively as compared to animals treated with AlCl_3_ alone.

The level of Aβ1-42 was significantly increased in the AlCl3-treated group by 362.5% compared to the control values. However, AlCl_3_ + BAs (125 mg/kg) or AlCl_3_ + BAs (250 mg/kg) significantly decreased Aβ1-42 levels when compared to AlCl_3_-treated group (Fig. 3B).

### Estimation of markers of oxidative stress and inflammation

It was found that AlCl_3_ injection caused an elevation in lipid peroxide formation (measured as MDA contents) that reached 955.2% as compared to the control group. However, BAs significantly decreased MDA in their two doses compared to the AlCl_3_-treated group. Levels of SOD and TAC of AlCl_3_-treated rats were significantly reduced by about 86% and 84%, respectively, compared to the control values. Animals treated with either dose of BAs exhibited a significant enhancement in SOD and TAC compared to AlCl_3_-treated animals (Table 1).

**Table 1:** Effect of Boswellic acids on Alzheimer’s-induced hippocampal oxidative stress and inflammation

Groups	Parameters				
	Oxidative stress markers			Inflammatory markers	
	MDA (ng/mg protein)	SOD (U/g tissue)	TAC (ng/g tissue)	TNF-α (pg/g tissue)	IL-1β (pg/g tissue)
Control (saline)	1.117 0.117	9.1 1.073	3.987 0.102	1.400 0.141	2.126 ± 0.117
AD	10.67 0.731^a^	1.31 0.143^a^	0.66 0.06^a^	11.72 0.81^a^	12.57 ± 0.98^a^
AD + BAs (125 mg/kg)	5.083 0.72^ab^	2.64 0.156^ab^	1.51 0.055^ab^	6.91 1.02 ^ab^	7.750 ± 1.01^ab^
AD + BAs (125 mg/kg)	4.06 0.234^abc^	4.1 0.228^abc^	2.275 0.11^abc^	4.71 0.232^abc^	5.68 ± 0.232^abc^

Data are mean ± SD (*n* = 6). a, b, or c: significantly different compared to the control, AD group or BAs (125 mg/kg) group respectively, *P* < 0.05 using ANOVA followed by Tukey’s as post hoc test

*AD* Alzheimer’s group (AlCl_3_ (100 mg/kg)), *BAs* Boswellic acids, *MDA* malondialdehyde, *SOD* superoxide dismutase, *TAC* total antioxidant capacity, *IL-1β* interleukin-1β, *TNF-α* tumor necrosis factor-α

Also, Table 1 displays hippocampal levels of TNF-α and IL-1β in the different treatment groups. AlCl_3_ showed marked increases in both TNF-α and IL-1β levels by 737.1% and 492%, respectively, compared to the control value. On the other hand, AlCl_3_ + BAs (125 mg/kg) or AlCl_3_ + BAs (250 mg/kg) groups modulated the increases in TNF-α and IL-1β tissue levels where they were decreased by (41.1%, 59.8%) and (38.4%, 54.8%) respectively as compared to AlCl_3_-treated group.

### Determination of the hippocampal levels of BDNF, pGSK-3β (Ser 9), and β-catenin

**Fig. 4:**
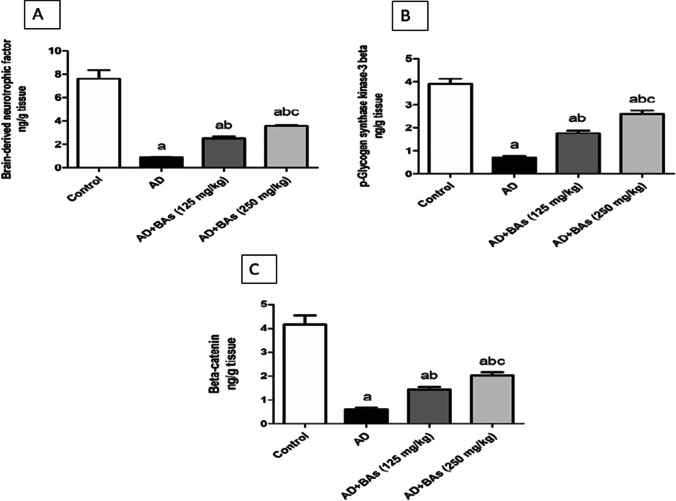
Effect of Boswellic acids on Alzheimer’s-induced alterations in the hippocampal brain-derived neurotrophic factor, p-glycogen synthase kinase-3 beta, and beta-catenin levels. **A** brain-derived neurotrophic factor, **B** p-glycogen synthase kinase-3 beta, **C** beta-catenin. Data are mean ± SD (*n* = 8). a, b, or c: significantly different compared to the control, AD group, or BAs (125 mg/kg) group respectively, *P* < 0.05 using ANOVA followed by Tukey’s as post hoc test. AD Alzheimer’s group (AlCl_3_ (100 mg/kg)), BAs Boswellic acids

To fulfill the underlying mechanisms incorporated in the neuroprotective effect of BAs, BDNF/GSK-3β/β-catenin axis was evaluated. Treatment with AlCl_3_ intensely reduced BDNF protein levels by 88.3% compared to the control levels. AlCl_3_-treated rats simultaneously administered either BAs (125 mg/kg) or BAs (250 mg/kg) showed a significant rise in BDNF levels by 183.1% and 302.1%, respectively, as compared to rats treated with AlCl_3_ alone (Fig. 4A). In addition, it was shown that AlCl_3_ injection significantly decreased the pGSK-3β (Ser 9) level by 81.9% compared to the control group. Nevertheless, AlCl_3_ + BAs (125 mg/kg) or AlCl_3_ + BAs (250 mg/kg) treatments significantly increased pGSK-3β (Ser 9) levels as compared to rats treated with AlCl_3_ only (Fig. 4B). Moreover, the effect of different treatment groups on β-catenin levels is displayed in Fig. 4C. AlCl_3_ exhibited a significant decrease in β-catenin level by 86% compared to the control group. However, in the two used doses, BAs significantly increased β-catenin levels compared to the AlCl_3_-treated group.

BAs (250 mg/kg) exhibited a more potent effect in all the measured biochemical parameters than BAs (125 mg/kg).

## Discussion

Recently, natural compounds have received much attention as an alternative or adjuvant therapy in treating NDs. Many of them have been traditionally used to improve learning and memory. Frankincense is the only recognized source of BAs responsible for the pharmacological properties of the gum resin of *Boswellia serrata*. This resin was described in ancient Ayurvedic scripts as a therapy for many inflammatory disorders. Lately, many studies have reported different pharmacological effects of the crude extract and BAs owing to their anti-inflammatory, antioxidant, anti-nociceptive, anti-bacterial, anti-arthritis, and neuroprotective properties (Al-Harrasi et al. 2019). Regarding its neuroprotective effects, traditionally, *Boswellia serrata* is recommended by Avicenna for pregnant women to improve the memory of their infants and for aged people to inhibit amnesia (Wynn and Fougère 2007).

The relationship between Al and AD was documented. Al deposits in different brain regions impairs memory and cognitive functions and boosts the deposition of Aβ, lipid peroxidation, and aggregation of hyperphosphorylated tau (Saba et al. 2017). In our study, the neurotoxic effects of AlCl_3_ were illustrated, where AlCl_3_-intoxicated rats showed increased AChE level with subsequent impairment of memory and cognition, augmented Aβ formation, induced oxidative stress, enhanced the expression of pro-inflammatory mediators and neuronal damage, as reported by Saba et al. (2017) and Justin Thenmozhi et al. (2017). Moreover, impairment of cognition, memory, and spatial learning was also recorded in the AlCl_3_-treated group. On the contrary, Bas-treated rats showed improvements in these behavioral abnormalities. Based on the literature, either *B. serrata* as an extract or isolated BAs can enhance memory and cognitive functions (Mahmoudi et al. 2011; Karima et al. 2012; Majdinasab et al. 2016; Ebrahimpour et al. 2017), which is explained based on the anti-inflammatory and antioxidant effects of BAs. In addition, decreased ACh level due to increased AChE activity participates in cognitive worsening. Accordingly, reduced AChE activity in BAs-treated groups was accompanied by improvements in abnormal behavior in OFT and MWM tests in harmony with Yassin et al. (2013) and Ebrahimpour et al. (2017).

Oxidative stress is the main contributor to the harmful effects of NDs. Therefore, the antioxidant treatment that reduces ROS is a promising key to decreasing AD progression. Lipid peroxide formation was elevated, while SOD and TAC levels were reduced in AlCl_3_-treated rats, as reported by Mohamed et al. (2021). Conversely, co-treatment with BAs restored the oxidative status. The antioxidant properties of BAs have been reported in different studies (Umar et al. 2014; Rajabian et al. 2020). A previous study by Ebrahimpour et al. (2017) said that BAs improve cognitive function and exhibit neuroprotective effects through their antioxidant action (Ebrahimpour et al. 2017).

Increased ROS production contributes to amyloid aggregation inside different brain regions. Aβ formation is the starting point for a long chain of pathophysiological actions in AD. Aβ aggregation activates excessive production of proinflammatory cytokines such as TNF-α and IL-1β, which also enhances a lot of Aβ overproduction as illustrated in our rat model. On the other hand, BAs significantly hinder Aβ aggregation and decrease TNF-α and IL-1β. The anti-amyloid effects of BAs can be explained in terms of their antioxidant and anti-inflammatory activities as neuroinflammation is considered one of the essential keys involved in NDs progression; drugs that have anti-inflammatory activities are supposed to treat or at least delay the progression. Our results agree with studies reporting the anti-inflammatory effect of BAs in different models (Shehata et al. 2011; Ammon 2019). Umar et al. (2014) said that *B. serrata* extract has an immune-modulatory effect and can be used to remedy chronic inflammatory diseases such as arthritis (Umar et al. 2014). Moreover, BAs reduce Aβ deposition and the cognitive dysfunction induced by lipopolysaccharide injection through its anti-inflammatory effect (Sayed and El Sayed 2016; Sayed et al. 2018).

The harmful effects of Aβ overexpression extend to reducing the expression of BDNF. BDNF is a master neurotrophin implicated in learning and memory, neurogenesis, synaptic plasticity, and dendritic density of the neurons. BDNF level is decreased in AD patients, accompanied by learning and memory impairment (Xie et al. 2017). So, to enhance memory and learning abilities, the BDNF level should be restored. The inhibition of proinflammatory mediators is also implicated in the neuroprotective property of BDNF (Fang et al. 2019). In our study, BAs reinstated BDNF level with subsequent enhancement of memory functions. The anti-amyloidogenic action of BAs may explain this result and its ability to hinder inflammation and oxidative stress. Despite traditional and pharmacological reports indicating the neuroprotective effects of *Boswellia* gum resin and its ability to enhance memory power, gaps are still present, specifically in the molecular mechanisms which underline its protective effects. It was reported by Hosseini et al. (2010) that treatment of rats with an aqueous extract of *Boswellia* enhances the spatial memory, and this effect is partly due to upregulation of BDNF and not via BDNF-CREB-BDNF cycle and suggests there is another pathway related to BDNF (Hosseini et al. 2010). It has been recently reported that BDNF applies its neurotrophic effects via crosstalk with the Wnt/β-catenin signaling pathway, and GSK-3β mediated this interaction and acted as the primary mediating crosstalk factor (Yang et al. 2016; Xie et al. 2017; Fang et al. 2019; Zhang et al. 2019). GSK-3β, a serine/threonine-protein kinase, controls many physiological functions. Its activity is regulated by phosphorylation with other proteins. Active GSK-3β is phosphorylated at (Tyr216), while phosphorylation at (Ser9) turns it inactive. Therefore, agents that can upregulate p-GSK-3β (Ser9) may be suggested to treat AD (Jaworski et al. 2019). GSK-3β, as a kinase, constantly phosphorylates different signaling molecules and transcription factors, one of these critical transcription factors is β-catenin. Phosphorylation of β-catenin by GSK-3β causes proteasomal degradation and discourages the translocation of β-catenin to the nucleus. Conversely, inhibition of GSK-3β catalytic activity allowed β-catenin to accumulate and migrate to the heart, where β-catenin encouraged de novo synthesis of essential growth factors and neurotrophins as BDNF (Yang et al. 2016; Libro et al. 2016). In addition, upregulation of BDNF enhances this pathway and inhibits GSK-3β activity (Yang et al. 2016). Furthermore, activation of Wnt signaling prevents Aβ production; in contrast, dysfunction of Wnt signaling promotes Aβ production and aggregation (Jia et al. 2019a). From the identified BDNF/GSK-3β/β-catenin axis, dysfunction of Wnt signaling is enough to encourage a neuropathological process incorporated in AD as cognitive impairment and aggregation of Aβ. Wnt/β-catenin signaling cascade is also a pathway for neuroprotection through suppression of oxidative stress, Aβ production, inflammation, and apoptosis (Xian et al. 2016; Vallée et al. 2017). Additionally, Wang et al. (2019) revealed that glutamine reduced oxidative stress-induced AD via activating the Wnt3/β-catenin signaling pathway. In the current study, the effect of BAs on the players in this cascade GSK-3β, BDNF, and β-catenin was investigated (Wang et al. 2019). In our rat AD model, we found that the p-GSK-3β (Ser9) level was decreased (i.e., increased GSK-3β activity) with a subsequent decrease in the total hippocampal β-catenin and BDNF levels. However, treatment with BAs upregulated GSK-3β (Ser9), which suppressed the activity of GSK-3β, increased β-catenin level, and enhanced BDNF production in the hippocampus. Regulation of the Wnt/β-catenin signaling cascade is not merely reflected in the memory and cognition (Hui et al. 2018) but also reflected in Aβ production and aggregation (Jia et al. 2019a), the inflammation, and oxidative status (Xian et al. 2016; Vallée et al. 2017) as explored in the current study. Up to date, few studies reported the effect of BAs on the Wnt/β-catenin signaling cascade, one regarding bone regeneration (Xiong et al. 2019) and others related to its anti-tumor activity (Liu et al. 2013). Recently, Gomaa et al. (2019) illustrated the effect of *B. serrata* on GSK-3β activity and its contribution to improving cognitive dysfunction in diabetic rats (Gomaa et al. 2019).

In conclusion, BAs have neuroprotective effects against AlCl_3_-induced AD symptoms, and this effect may be mediated, to some extent, through the anti-amyloidogenic, anti-inflammatory, and antioxidant activities of BAs. The probable mechanisms underlying the potential neuroprotective activity of BAs may also be attributed to their modulatory effect of the Wnt/β-catenin signaling cascade.

## Supplementary Information

Below is the link to the electronic supplementary material.

Supplementary file1 (JPEG 60 KB)

Supplementary file2 (MP4 6355 KB)

Supplementary file3 (MP4 2594 KB)

Supplementary file4 (MP4 11537 KB)

Supplementary file5 (MP4 16954 KB)

## Data Availability

The authors confirm that the data supporting the findings of this study are available within the article.

## Abbreviations

Aβ: Amyloid-β
AD: Alzheimer’s disease
BA: Bosweillic acids
BDNF: Brain-derived neurotrophic factor
NDs: Neurodegenerative diseases
GSK-3β: Glycogen synthase kinase 3β
