# Supplementary figures and images for: Boswellic acids ameliorate neurodegeneration induced by AlCl3: the implication of Wnt/β-catenin pathway
Source: Environ Sci Pollut Res Int. 2022 Jun 6;29(50):76135–43. doi: 10.1007/s11356-022-20611-5 (PMC9553772; doi:10.1007/s11356-022-20611-5)

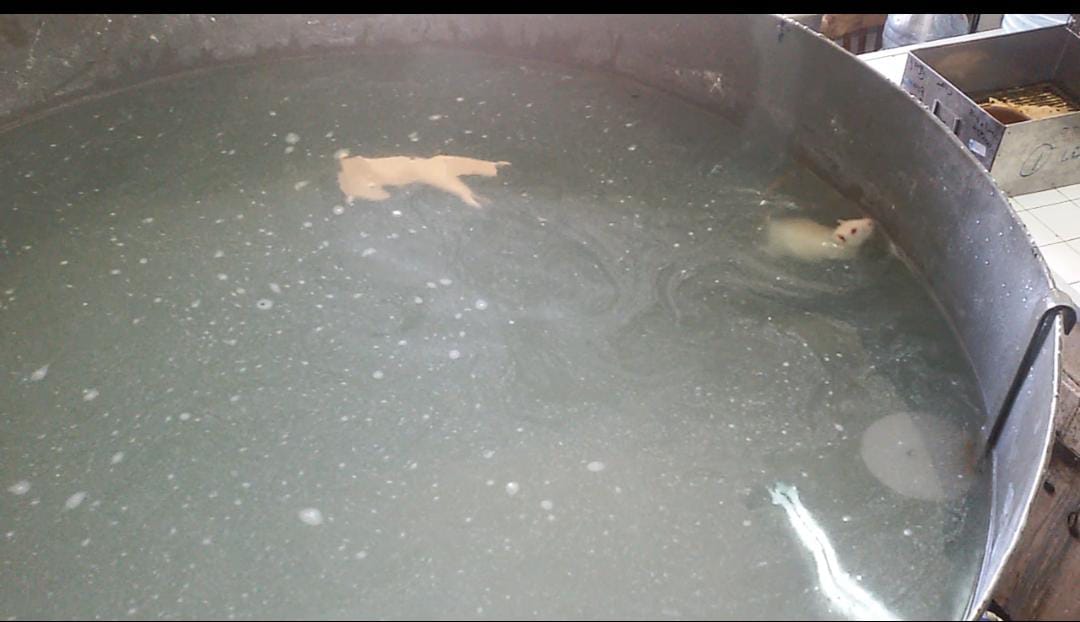

Supplement: Supplementary file 1 — Supplementary file1 (JPEG 60 KB) [file 11356_2022_20611_MOESM1_ESM.jpeg]
